# Long noncoding RNA#45 exerts broad inhibitory effect on influenza a virus replication via its stem ring arms

**DOI:** 10.1080/21505594.2021.1975494

**Published:** 2021-09-14

**Authors:** Lei Zhang, Xinxin Zheng, Jun Li, Guoqing Wang, Zenglei Hu, Yu Chen, Xiaoquan Wang, Min Gu, Ruyi Gao, Shunlin Hu, Xiaowen Liu, Xinan Jiao, Daxin Peng, Jiao Hu, Xiufan Liu

**Affiliations:** a Animal Infectious Disease Laboratory, School of Veterinary Medicine, Yangzhou University, Yangzhou, China; b Jiangsu Co-innovation Center for Prevention and Control of Important Animal Infectious Diseases and Zoonosis, Yangzhou University, Yangzhou, China; c Key Laboratory of Prevention and Control of Biological Hazard Factors (Animal Origin) for Agri-food Safety and Quality, Ministry of Agriculture of China (26116120), Yangzhou University, Yangzhou, China; d Jiangsu Key Laboratory of Zoonosis, Yangzhou University, Yangzhou, China

**Keywords:** Long non-coding RNA, influenza A virus, replication, antiviral strategy, RNA-FISH

## Abstract

A growing body of evidence suggests the pivotal role of long non-coding RNA (lncRNA) in influenza virus infection. Based on next-generation sequencing, we previously demonstrated that Lnc45 was distinctively stimulated by H5N1 influenza virus in mice. In this study, we systematically investigated the specific role of Lnc45 during influenza A virus (IAV) infection. Through qRT-PCR, we first demonstrated that Lnc45 is highly up-regulated by different subtypes of IAV strains, including H5N1, H7N9, and H1N1 viruses. Using RNA-FISH and qRT-PCR, we then found that Lnc45 can translocate from nuclear to cytoplasm during H5N1 virus infection. In addition, forced Lnc45 expression dramatically impeded viral replication of H1N1, H5N1, and H7N9 virus, while abolish of Lnc45 expression by RNA interference favored replication of these viruses, highlighting the potential broad antiviral activity of Lnc45 to IAV. Correspondingly, overexpression of Lnc45 inhibited viral polymerase activity and suppressed IAV-induced cell apoptosis. Moreover, Lnc45 significantly restrained nuclear aggregation of viral NP and PA proteins during H5N1 virus infection. Further functional study revealed that the stem ring arms of Lnc45 mainly mediated the antiviral effect. Therefore, we here demonstrated that Lnc45 functions as a broad-spectrum antiviral factor to inhibit influenza virus replication probably through inhibiting polymerase activity and NP and PA nuclear accumulation via its stem ring arms. Our study not only advances our understanding of the complexity of the IAV pathogenesis but also lays the foundation for developing novel anti-IAV therapeutics targeting the host lncRNA.

## INTRODUCTION

Influenza A virus (IAV) is an enveloped negative-strand RNA virus that can be classified into type A, B, and C according to different antigenicity of the nucleoprotein (NP) and matrix protein (M) [1]. IAV has a wide host range and poses a dual threat to both poultry and human health [2].

Long non-coding RNA (lncRNA) is a large class of non-protein-coding transcripts with a length of more than 200 nucleotides (nt) [3]. Although lncRNA does not contain a significant open reading frame, it can act as a gene regulator at multiple levels, including transcription, post-transcription and translation [4–7]. Moreover, accumulated studies have demonstrated that lncRNA plays either a promotional or an inhibitory role in the process of viral infection [8–11]. For example, lncRNA NRON (noncoding repressor of Nuclear Factor of Activated T cells [NFAT]), modulates human immunodeficiency virus (HIV) replication through alternating the activity of NFAT and the viral long terminal repeat (LTR) region [9]. While LncRNA GAS5 (growth arrest-specific 5), which was first identified as a decoy of the hepatitis C virus (HCV) NS3 protein, is important for preventing HCV replication in Huh7 cells [10]. In addition, lncRNA also represents a defender of host immune response to viral infection [12–14]. For instance, Lnczc3h7a serving as a molecular scaffold between the E3 ubiquitin ligase TRIM25 and RIG-I, then facilitates the subsequent innate immune signaling and promising an efficient antiviral effect [14].

As for influenza virus, lncRNA also plays a substantial role during influenza virus infection and contributes to the antiviral immune response [15–22]. In order to directly or indirectly regulate IAV replication, abundant differentially expressed lncRNAs are induced by IAV infection to play important roles in immune signal transduction, immune pathway activation and the generation of antiviral proteins [22]. Some previous studies emphasize the functions of lncRNA, either being as negative or positive regulator in influenza virus replication or functional as a regulator in innate antiviral immunity, including NRAV (negative regulator of antiviral response), lnc-ISG20, lncRNA ISR, VIN (virus inducible lncRNA), lncRNA TSPOAP1-AS1 and lncRNA-155 [15–21]. NRAV is one of such examples, which impedes the expression of some critical interferon-stimulated genes (ISGs), such as IFITM3 (interferon-induced transmembrane protein 3) and MxA, thus significantly promotes IAV replication and viral virulence [21]. In contrast, lnc-ISG20, which is a novel interferon-stimulated gene stimulated by IAV, exerts its antiviral effect via up-regulating the expression of ISG20 [20]. Moreover, for function of lncRNA in innate immunity, lncRNA TSPOAP1-AS1 inhibits IAV-induced IFN-β1 transcription, impedes the activation of interferon-sensitive response element (ISRE), and suppresses the expression of the downstream interferon-stimulated gene [17]. Besides, IAV-induced lncRNA-155, which was encoded by MIR155HG, modulates immune response via regulation of PTP1B (Protein Tyrosine Phosphatase 1B)-mediated interferon response during influenza virus infection [15].

Through deep-sequencing of the different lncRNA expression during H5N1 influenza virus infection in mice, we previously identified a potential functional lncRNA, named Lnc45 [23]. Here, we demonstrated that Lnc45 was highly stimulated by various subtypes of influenza A virus, including H5N1, H1N1, and H7N9, and exerts a wide inhibitory effect on viral replication of various subtypes of IAV. Furthermore, Lnc45 translocated from nuclear to cytoplasm after IAV infection. Over-expression of Lnc45 significantly inhibited viral polymerase activity, impeded nuclear aggregation of NP and PA proteins and suppressed IAV-induced cell apoptosis. Moreover, the broad inhibitory antiviral effect was mainly mediated by the stem ring arms of Lnc45. Therefore, our results demonstrated that Lnc45 was equipped with the broad-spectrum anti-influenza A virus activity that may act as potential antiviral complements for IAV infection.

## MATERIALS AND METHODS

### Bioinformatics analysis

The University of California Santa Cruz (UCSC) Genome Browser (http://genome-asia.ucsc.edu/index.html) was explored to predict the location of Lnc45 on chromosome of the mouse genome. The ability of Lnc45 to encode gene was predicated through PhyloCSF tracks (https://data.broadinstitute.org/compbio1/PhyloCSFtracks/trackHub/hub.txt). In order to analyze the secondary structure of Lnc45, an RNAfold web server (http://rna.tbi.univie.ac.at/cgi-bin/RNAWebSuite/ RNAfold.cgi) was also made use of. The potential transcription factor (TF) of Lnc45 was predicted through TRANSFAC (http://www.gene-regulation. Com/index2.html).

### Virus strains and Cells

Influenza A viruses, including A/Chicken/Jiangsu/k0402/2010 (H5N1 CK10) [23], A/Puerto Rico/8/1934 (H1N1 PR8) and A/Chicken/Shandong/S8/2015 (H7N9 S8) were propagated in specific pathogen free (SPF) embryonated chicken eggs at 37°C for about 1 to 2 days.

Human embryonic kidney cells (HEK293T/293 T), Madin–Darby canine kidney cells (MDCK) cells, Rat alveolar type II cells (RLE-6TN) and Henrietta Lack cell (HeLa) were cultured in Dulbecco’s Modified Eagle’s Medium (DMEM) (Gibco, MA, USA) supplemented with 10% heat-inactivated fetal bovine serum (FBS) (Gibco), and then incubated in a 37°C, 6% CO_2_ incubator.

### Plasmids and siRNA

The Lnc45 gene was subcloned into the expression plasmid pcDNA3.1. Plasmids expressing the PB2, PA, PB1, and NP genes of the CK10 virus were generated previously by subcloning of these genes into the pcDNA3.1 vector [24]. The Firefly luciferase reporter plasmid and the internal control *Renilla* luciferase reporter plasmid were from our laboratory.

In order to obtain the truncation mutants of Lnc45, the truncation mutants were PCR-amplified using the cDNA of the Lnc45 as a template and then were subcloned into expression plasmid pcDNA3.1 using a Ready-to-Use Seamless Cloning Kit (Sangon Biotech, Shanghai, China) according to the manufacturer’s protocol. The mutated plasmids were named Mutant-A to Mutant-L, respectively (Figure 11a). All plasmids constructed in this study were confirmed by sequencing, and the primers used are shown in **Table S1**.

**Figure 1.**
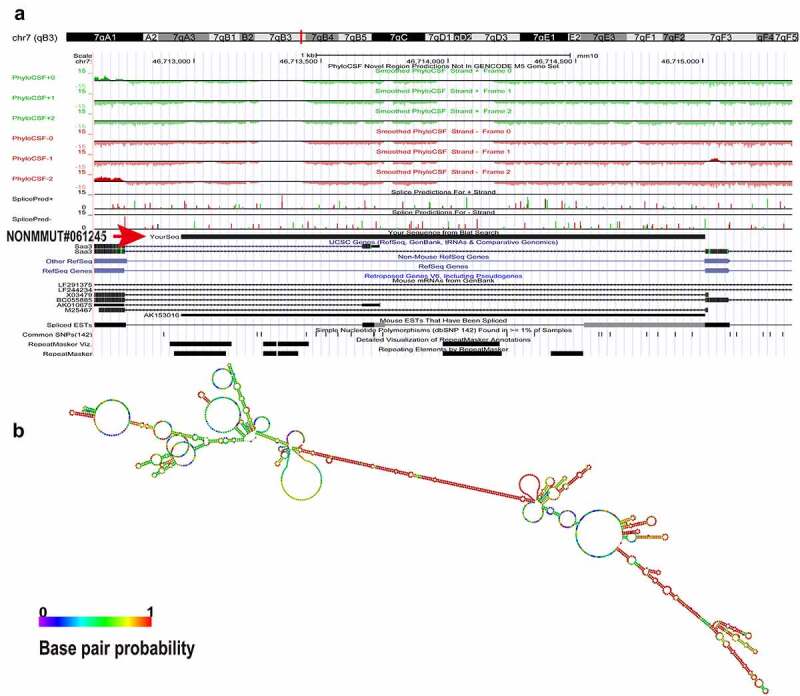
**Bioinformatics analysis of Lnc45**. (a) The UCSC database was used to predict the chromosome location of Lnc45 in the mouse genome. The ability of Lnc45 to encode gene was predicated through PhyloCSF tracks. (b) RNA secondary structure for Lnc45 was analyzed using RNAfold (RNAfold web server, University of Vienna). The data was shown as a minimal free energy structure (MFE = −396.90 kcal/mol). Base pairing probabilities have been color-coded on a scale from 0 (blue) to 1 (red)

**Figure 2.**
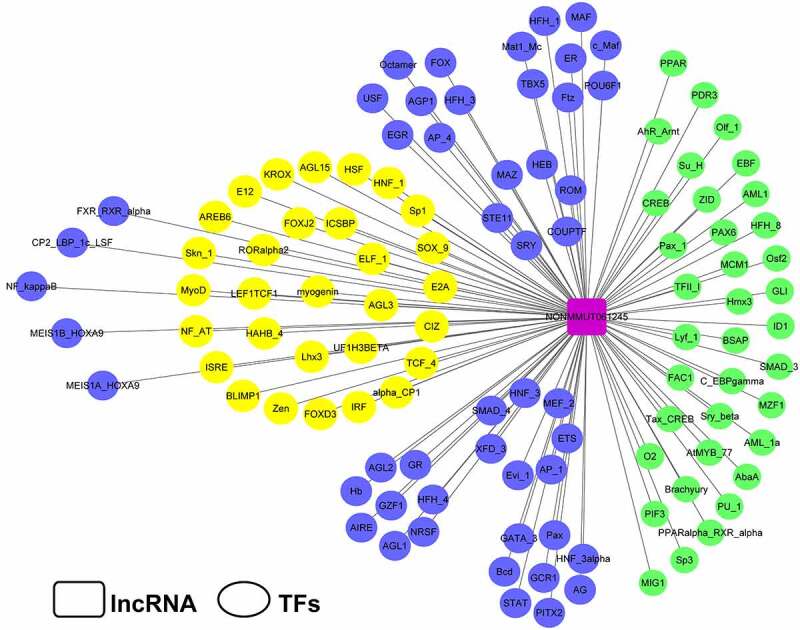
**Transcriptional factor analysis of Lnc45**. The potential transcriptional factors (TFs) of Lnc45 were analyzed via TRANSFAC database

**Figure 3.**
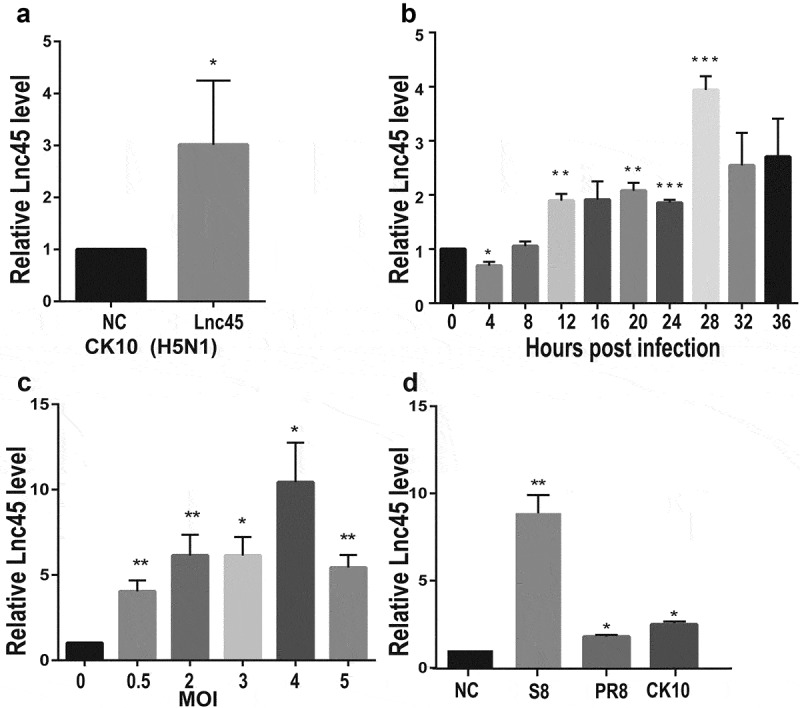
**Lnc45 is highly stimulated by different subtypes of IAV strains**. (a) 6TN cells were infected with 1 MOI of H5N1 IAV (CK10) for 24 h. Expression level of Lnc45 was determined by qRT-PCR. (b) Expression levels of Lnc45 in 6TN cells after infected with 1 MOI of CK10 virus was determined by qRT-PCR according to different infection time course. (c) 6TN cells were infected with CK10 virus at different MOIs for 24 h. Expression level of Lnc45 was determined by qRT-PCR. (d) 6TN cells were infected with different subtypes of influenza virus, including H5N1 (CK10), H7N9 (S8) and H1N1 (PR8), in a dose of 1 MOI for 24 h. Lnc45 expression by qRT-PCR was shown as mean fold-changes ± standard deviation (SD) compared with non-infected reference (NC). Data from three independent experiments were examined using one-sample t-test (* P < 0.05; ** P < 0.01; *** P < 0.001)

**Figure 4.**
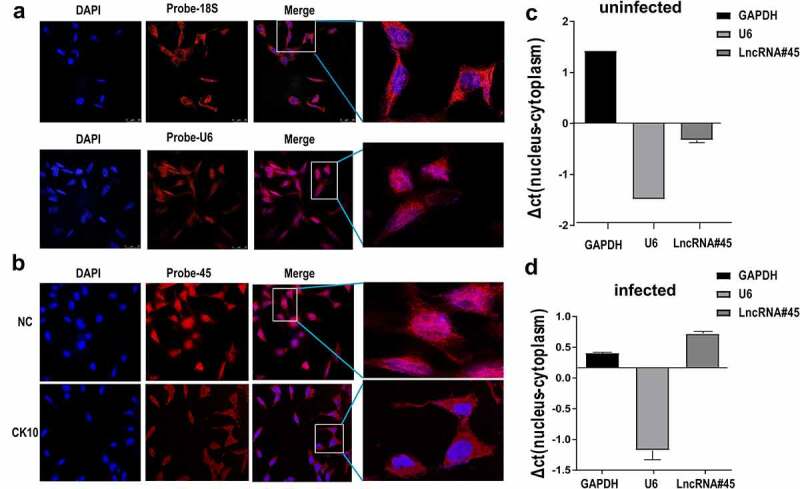
**Lnc45 is translocated from nuclear to cytoplasm during H5N1 IAV infection**. (a) Nuclei was visualized using DAPI (blue). 18S and U6 probes were hybridized 6TN cells which shown as red immunofluorescence. Images shown are representatives from three independent experiments. (b) FISH and confocal imaging showed the localization of Lnc45 (red) in the cell nucleus 6TN cells before or after CK10 virus infection. (c) Nuclear and cytoplasmic RNA fractions were separated from uninfected 6TN cells. qRT-PCR was performed to determine the expression of the GAPDH, U6 and Lnc45. (d) Nuclear and cytoplasmic RNA fractions were isolated from CK10 virus-infected 6TN cells (1 MOI, 24 h). GAPDH, U6 and Lnc45 expression by qRT-PCR was shown as mean fold-changes ± SD compared with non-infected reference (NC)

**Figure 5.**
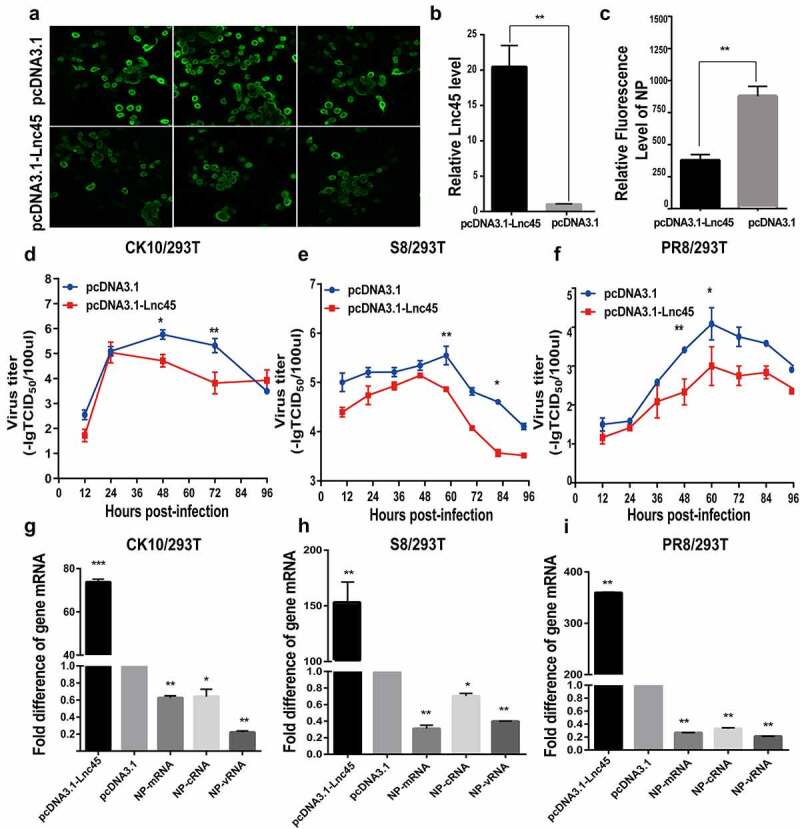
**Over-expression of Lnc45 efficiently inhibits viral replication of different subtypes IAV in 293 T cells**. (a) 293 T cells were transfected with pcDNA3.1-Lnc45 or pcDNA3.1 and then infected with the CK10 virus (1 MOI, 24 h). Expression of viral NP gene (green) was analyzed by the indirect immunofluorescence assay. (b) The efficiency of over-expression of Lnc45 in 293 T cells was evaluated through qRT-PCR. (c) The relative level of NP fluorescence intensity compared with pcDNA3.1 group was determined by ImageJ Plus. (d-f) 293 T cells were first transfected with pcDNA3.1-Lnc45 or pcDNA3.1 for 24 h, and then the cells were inoculated with CK10 (d), S8 (e) and PR8 (f) virus (1MOI, 24 h), respectively. The cellular supernatants were then collected at indicated time points post infection and viral load was quantified based on TCID_50_ on MDCK cells. (g-i) 293 T cells were first transfected with pcDNA3.1-Lnc45 or pcDNA3.1 for 24 hours, and followed by infection with CK10 (d), S8 (e) or PR8 (f) virus (1 MOI) for 24 h, respectively. The total RNA was extracted from cells collected at indicated time points post-IAV infection. Then the levels of Lnc45 and viral NP mRNA, cRNA, vRNA were determined by qRT-PCR. The data was shown as the means ± SD for triplicate samples from one representative independent experiment. The experiments were replicated three times and analyzed using one-sample t test. * P < 0.05, ** P < 0.01 and *** P < 0.001, shown as significant different compared with the result of the pcDNA-3.1 control group

**Figure 6.**
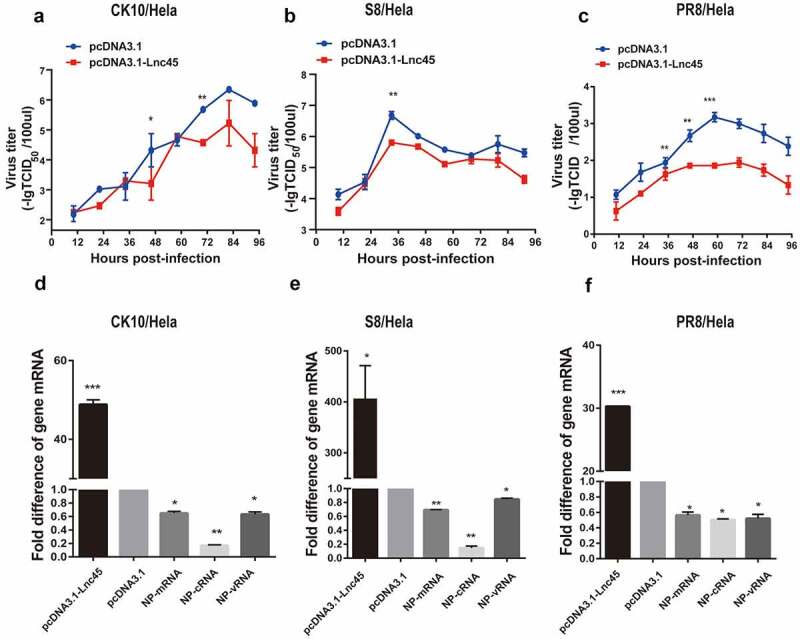
**Over-expression of Lnc45 efficiently inhibits viral replication of different subtypes IAV in Hela cells**. (a-c) Hela cells were transfected with pcDNA3.1-Lnc45 or pcDNA3.1 and then exposed to CK10 (a), S8 (b) or PR8 (c) virus infection (1MOI, 24 h), respectively. The virus titers in cellular supernatants collected at indicated time points were measured based on the TCID_50_ in MDCK cells. (d-f) Hela cells were transfected with pcDNA3.1-Lnc45 or pcDNA3.1 for 24 h and then were inoculated with CK10 (d), S8 (e) or PR8 (f) (1 MOI, 24 h) virus, respectively. The expression levels of viral NP mRNA, cRNA, vRNA and Lnc45 in infected Hela cells were measured using qRT-PCR. The data was shown as the means ± SD for triplicate samples from one representative independent experiment. * P < 0.05, ** P < 0.01 and *** P < 0.001, shown as significant different compared with the result of the pcDNA-3.1 control group

**Figure 7.**
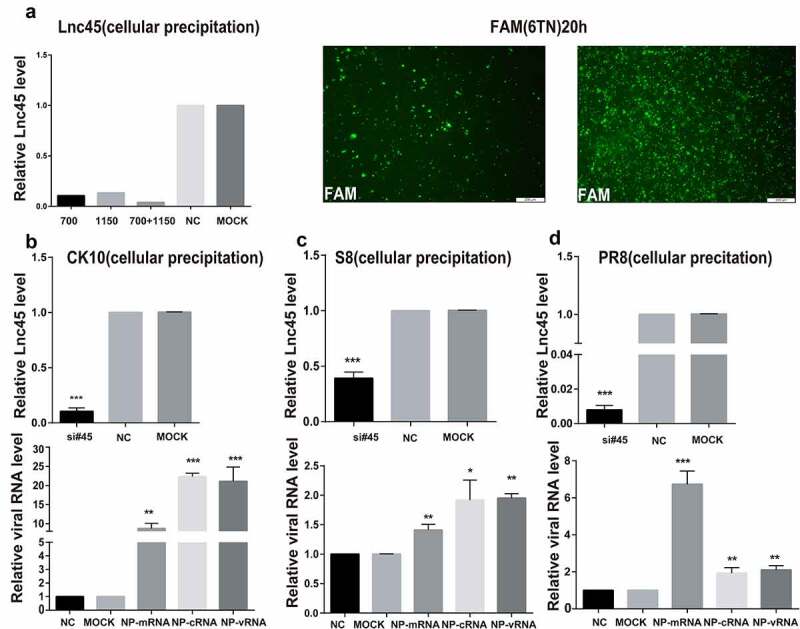
**Knockdown Lnc45 highly promotes viral replication of different subtypes IAV in 6TN cells (cellular precipitation). (a left panel)** 6TN cells were transfected with siRNA700 or siRNA1150, respectively or co-transfected with siRNA700 and siRNA1150. The interference efficiency of Lnc45 was measured using by qPT-PCR. **(a right panel)** Transfection efficiency of 6TN cells was determined through FAM (green) fluorescence intensity after 20 h. **(b, c, d upper panel)** 6TN cells were co-transfected with siRNA700 and siRNA1150 for 24 h, and then followed by infection with different subtypes IAV (CK10 (b), S8 (c) and PR8 (d)) virus (1MOI, 24 h), respectively. The interference efficiency of Lnc45 was measured using by qPT-PCR. **(b, c, d lower panel)** The total RNA of 6TN cellular precipitation was extracted for qRT-PCR at 24 h pi. Viral NP mRNA, cRNA, vRNA expression was normalized to GAPDH using the 2^−ΔΔCt^ method. The data represents the means ± SD of three independent experiments. * P < 0.05, ** P < 0.01 and *** P < 0.001, shown as significant different compared with the result of the mock control cells

**Figure 8.**
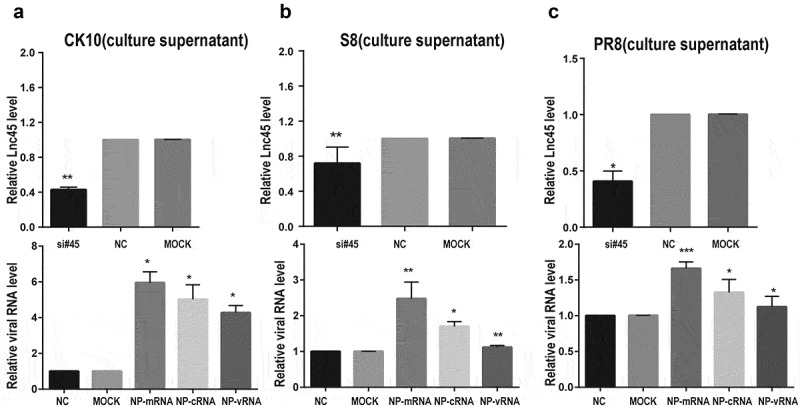
**Knockdown Lnc45 highly promotes viral replication of different subtypes IAV in 6TN cells (cellular supernatants). (a, b, c upper panel)** 6TN cells were co-transfected with siRNA700 and siRNA1150 for 24 h. Then, the expression of levels of Lnc45 were detected by qRT-PCR. **(a, b, c lower panel)** The 6TN cells were infected with CK10 (a), S8 (b) and PR8 (c) virus at an MOI of 1 for 24 h, respectively. The total RNA of the cellular supernatants was extracted for qRT-PCR at 24 h p.i. Viral NP mRNA, cRNA, vRNA expression was normalized to GAPDH using the 2^−ΔΔCt^ method. The data represents the means ± SD of three independent experiments. * P < 0.05, ** P < 0.01, and *** P < 0.001, shown as significant different compared with the result of the mock control cells

**Figure 9.**
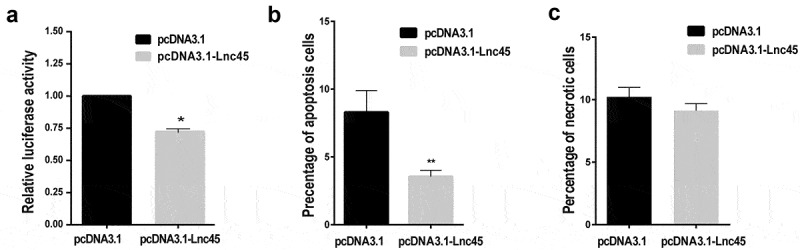
**Over-expression of Lnc45 significantly inhibits viral polymerase activity and cell apoptosis**. (a) 293 T cells were transfected with pcDNA3.1-Lnc45 or pcDNA3.1, then followed by co-transfection of RNP reconstitution plasmids (PB1, PB2, PA, NP) and the reporter plasmid that used to transcribe an IAV-like RNA. After 24 h, cell lysates were used to measure luciferase activities. The polymerase activity values were normalized to the Renilla luciferase activity. (b and c) 293 T cells were first transfected with pcDNA3.1-Lnc45 or pcDNA3.1 for 24 h, and then the cells were inoculated with CK10 virus (1 MOI, 24 h). The 293 T cells were collected at indicated time points and then analyzed for cell apoptosis (b) and necrosis (c) using flow cytometry. The experiments were carried out in triplicate and repeated three times, and the data are expressed as mean ± SD of three independent experiments. All statistical analyses were carried out using the t-test. * P < 0.05 and ** P < 0.01, shown as significant different compared with the result of the mock control cells

**Figure 10.**
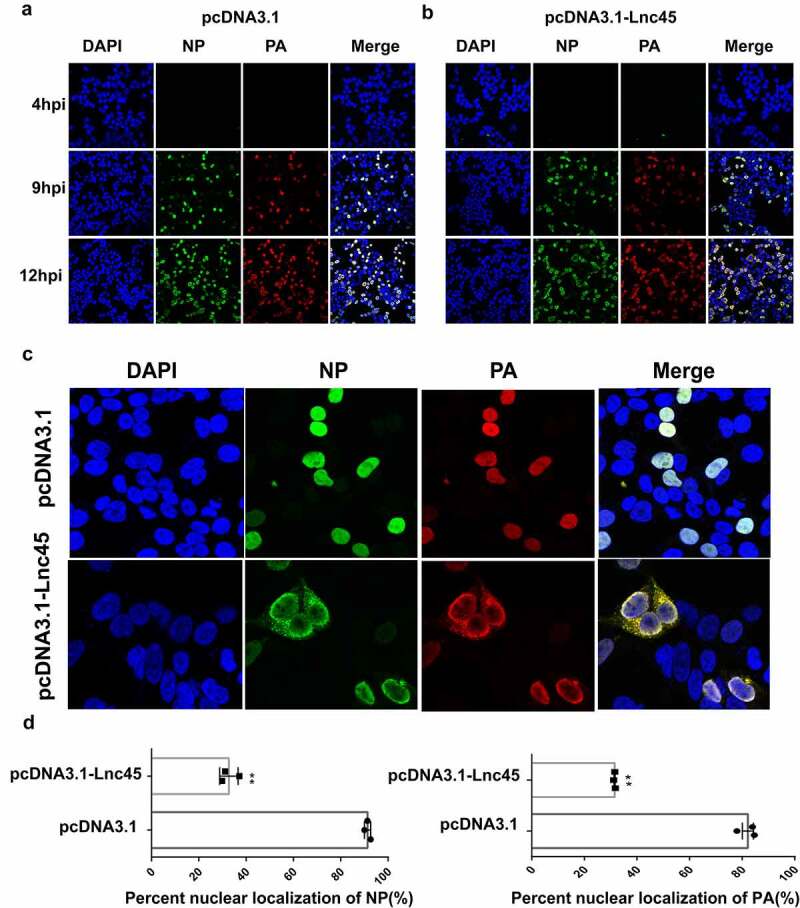
**Over-expression of Lnc45 down-regulates PA and NP nuclear accumulation**. (a, b) 293 T cells were first transfected with pcDNA3.1-Lnc45 or pcDNA3.1 for 24 h, then the cells were inoculated with CK10 virus (1 MOI, 4 h, 9 h or 12 h). 293 T cell cultures were then fixed and processed for immunofluorescence observation at the indicated time points. Cell nuclei were stained with DAPI. (c) 293 T cells were transfected with pcDNA3.1or pcDNA3.1-Lnc45 and then infected with CK10 virus at an MOI of 1. At 9 h p.i., cells were fixed and processed for immunofluorescence observation. (d)The PA and NP nuclear accumulation in the infected cells was determined as the ratio of cells showing red (PA) or green (NP) fluorescence in the nucleus to the total number of cells counted (n = 200). The values shown are the means ± SD of the results from three independent experiments. ** P < 0.01, shown as significant different compared with the result of the control cells. Three independent replicates were set up for each independent experiment. Moreover, three areas were randomly selected from each replicate well, and a total of 200 cells in each area were analyzed. Therefore, 1800 cells (3*3*200 cells) were statistically analyzed in total in each independent experiment

**Figure 11.**
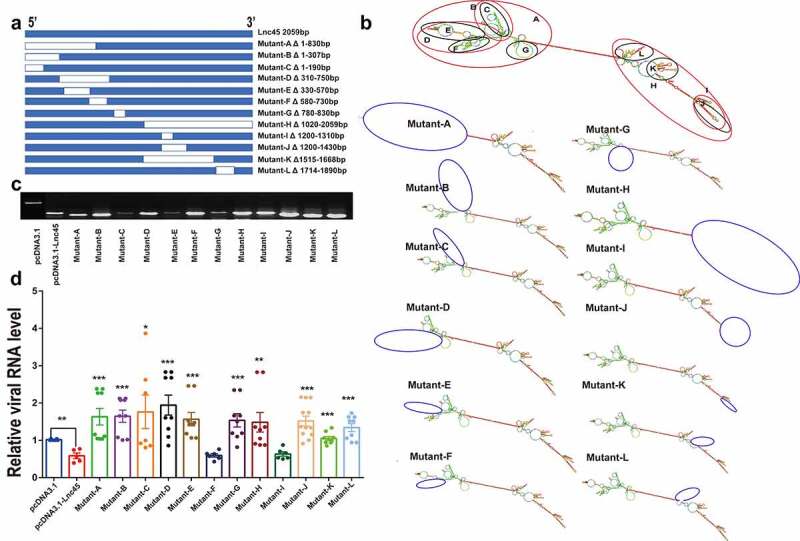
**The stem ring arms mainly determine the antiviral effect of Lnc45**. (a) Schematic diagram of truncation mutants of Lnc45 was shown. (b) Secondary structure predictions of Lnc45 and the Lnc45 mutants were performed through RNAfold. The mutation locations were labeled by blue circle. (c) Exogenous expression of Lnc45 or its mutants in 293 T cells was determined by RT-PCR. (d) 293 T cells expressing Lnc45 or its mutants were infected with IAV, and the mRNA of virus protein (NP) was demonstrated by qRT-PCR. * P < 0.05, ** P < 0.01, *** P < 0.001, means significant different compared with the parental plasmid pcDNA-3.1-Lnc45. Shown are means ± SD of representative results from three independent experiments

For silencing of Lnc45 expression, Lnc45-specific siRNAs were transfected into 6TN cells using siRNA-Mate (Gene Pharma, SuZhou, China) following the supplied protocol. Moreover, the negative-control siRNAs and FAM-siRNA were also included. The specific sequences of the used siRNAs are given in Table 1.

**Table 1.** siRNA sequences (5′–3′)

Gene	sense	antisense
siRNA-1150	GGUCCUUAGUUUAGAUCUUTT	AAGAUCUAAACUAAGGACCTT
siRNA-700	CCUGGGACAUAGGUCUAUUTT	AAUAGACCUAUGUCCCAGGTT
FAM-siRNA	UUCUCCGAACGUGUCACGUTT	ACGUGACACGUUCGGAGAATT
N.C siRNA-RL	GCGACGAUCUGCCUAAGAUdTdT	AUCUUAGGCAGAUCGUCGCdTdT

### Cell death analysis

293 T cells were first transfected with pcDNA3.1-Lnc45 or pcDNA3.1 for 24 h, and then the cells were then inoculated with CK10 virus. At 24 h p.i, the cells were then collected and analyzed as described previously [25]. Briefly, a total of 10^6.0^ cells was stained with fluorescein isothiocyanate (FITC)-conjugated annexin V and/or propidium iodide (PI) (Beyotime, Shanghai, China), respectively, and were then analyzed using the flow cytometer (Becton Dickinson, Franklin Lakes, NJ, USA). The ratios of apoptotic or necrotic cells were presented as the means ± standard deviations (SD) from three independent experiments.

### Virus Growth curve

To determine whether Lnc45 affect virus replication, 293 T cells and Hela cells with 80–90% confluence was first transfected with pcDNA3.1-Lnc45 or pcDNA3.1 for 24 h, then the cells were infected with indicated influenza virus at a multiplicity of infection (MOI) of 1. The supernatants of the cell culture were harvested at the indicated times points. The virus 50% tissue culture infective dose (TCID_50_) in supernatants were measured on MDCK cells by the method of Reed and Muench [26].

### Indirect immunofluorescence assay

For indirect immunofluorescence assay, cells were washed three times using PBS, then fixed in 4% paraformaldehyde at room temperature for 30 min, and permeabilized in 0.5% Triton X-100 for 10 min. The cells were then blocked with 1% bovine serum albumin (BSA) for 1 h at room temperature, and were incubated with rabbit antiserum against PA (1:1000, GeneTex, San Antonio, USA) or monoclonal antibody against NP (1:300, generated by our lab) overnight at 4°C. PBS was used to wash the cells for additional three times and incubated for 1 h with Alexa-conjugated goat anti-rabbit (for PA) or fluorescein isothiocyanate (FITC)-coupled goat anti-mouse (for NP) secondary antibodies (1:1000, Invitrogen, CA, USA) at room temperature. The nucleus was stained with DAPI (4,6-diamino-2-phenyl indole). Fluorescence was then observed by a Leica TCS SP-E microscope.

### Subcellular Fractionation

6TN cells that were cultured in a six-well plate were washed with PBS and incubated with 200 µL buffer A (10 mM HEPES, 10 mM KCl, 1.5 mM MgCl2, 0.34 M sucrose, 10% glycerol, 1 mM DTT, 0.1% Triton X-100 and protease inhibitor) for 5 min on ice, followed by low-speed centrifugation at 4 ◦C (1500 × g, 4 min). The supernatant was then further clarified by high-speed centrifugation (13,000 × g, 10 min) to remove cell debris and insoluble aggregates, and then stored as the cytoplasmic fraction. The pellets were then washed once with buffer A without 0.1% Triton X-100, then lysed in 200 µL RIPA buffer (50 mM Tris, pH 7.4, 150 mM NaCl, 1% Triton X-100, 1% sodium deoxycholate, 0.1% SDS and protease inhibitor), and then stored as nuclear fraction. For RNA quantification in the cytoplasm and nuclei, the subcellular fractions of 6TN cells were prepared in the presence of RNase inhibitor as described above. The levels of Lnc45, GAPDH and U6 mRNA in cytoplasmic and nuclear fractions were then determined by quantitative real-time PCR (qRT-PCR). The percentage of RNA distribution was determined as the ratio of the amount of RNA in each fraction to the total amount of RNA.

### qRT-PCR

To determine the amount of Lnc45 and NP mRNA, cRNA and vRNA after virus infection, the total RNA for the uninfected or infected 293 T cells were collected using TRIZOL (Vazyme, NanJing, China) following the manufacturer’s instructions. To determine the Lnc45 expression in the nuclear and cytoplasm, the total RNA for the cytoplasmic and nuclear fractions of the uninfected or infected 6TN cells were collected separately using TRIZOL (Vazyme) following the manufacturer’s instructions. Reverse transcription reactions were performed with the Prime Script RT reagent Kit (TaKaRa). The qRT-PCR was then performed with the SYBR Premix ExTaq Kit (TaKaRa) according to the manufacturer’s protocol. U6 mRNA was measured as nuclear endogenous control and GAPDH mRNA was measured as cytoplasm endogenous control. The final results were analyzed by normalizing against GAPDH or U6 using the 2^−ΔΔCT^ method. The specific primer sequences have been listed in Table 2.

**Table 2.** Primers used for qRT-PCR detection

Primers	Primer sequence (5′–3′)
CK10 NP-F	AGAGACGGAAAATGGGTGAGAGAGC
CK10 NP-R	GGATCCATTCCAGTACGCACGAGAG
S8 NP-F	AGAGACGGAAAATGGGTGAGAGAGC
S8 NP-R	GGATCCATTCCAGTACGCACGAGAG
PR8 NP-F	GCGTCTCAAGGCACCAAAC
PR8 NP-R	TCAAAAGCAGAGAGCACCATTC
NP-vRNA	AGCAAAAGCAGGGTAGATAATCACTC
NP-cRNA	AGTAGAAACAAGGGTATTTTTCTTT
Lnc45-F	GAGCATTTTCCACGGACTTC
Lnc45-R	AAACCCCTACCTCCTCTCCA
(Human) GAPDH-F	GGTGGTCTCCTCTGACTTCAACA
(Human) GAPDH-R	GTTGCTGTAGCCAAATTCGTTGT
(Mus) GAPDH-F	CGTGTTCCTACCCCCAATGT
(Mus) GAPDH-R	TGTCATCATACTTGGCAGGTTTCT
(Mus) U6-F	GCTTCGGCAGCACATATACTAAAAT
(Mus) U6-R	CGCTTCACGAATTTGCGTGTCAT

## RNA-FISH

A fluorescent *in situ* hybridization (FISH) Kit (RiboBio, Guangzhou, China) was used to determine the distribution of Lnc45 in 6TN cells. The 6TN cells were seeded in dishes with coverslips and infected with CK10 virus for 24 h. Cells were then fixed in 4% paraformaldehyde at room temperature for 30 min, and then washed three times with PBS, and permeabilized with 0.5% Triton X-100 on ice for 10 min. Following additional three times washes in PBS for 10 min; cells on coverslips were pre-hybridized at room temperature for 30 min. The probe for U6 and 18S mRNA were used as control for FISH assay. The 2× saline sodium citrate (SSC) was used to cover the treated cells. Then, the cells were heated at 95°C for 4 min, and incubated at 37°C overnight in a humidified chamber containing denatured probes and the supplemented hybridization buffer (20% formamide, 10% dextran sulfate, 10% 20× SSC, 100 g yeast tRNA, 0.5 μl rRNasin). Coverslips were then washed with 2× SSC/50% formamide (three times) at 37°C, 2× SSC (three times) at 42°C, 1× SSC (three times) at 42°C, and 4× SSC (once) at room temperature. The cell nucleus was stained with DAPI. A Leica TCS SP-E microscope was used to analyze the confocal immunofluorescence images.

### Dual-luciferase assay

293 T cells cultured on 12-well plate were co-transfected with 200ng/well of pcDNA3.1 encoding PB1, PB2, PA, and NP genes from CK10 virus and a Firefly luciferase reporter plasmid (p-Luci), or an empty plasmid , together with 20ng/well ofa internal control *Renilla* plasmid (pTK-RL)  and 250 ng of Lnc45 using EL Transfection Reagent (Roche). After 24 h, luciferase activities were determined using the Dual-GLO® Luciferase Assay System Kit (Promega, Madison, WI, USA) following the supplied instruction. Results of the polymerase activity were calculated as relative luciferase activity which presented as the ratio of Firefly luciferase/Renilla luciferase. Results are shown as the means ± standard deviations (SD) from three independent experiments.

### Statistical analysis

Statistical analyses were performed using the independent-samples *t* based on SPSS statistics software. “*” (P < 0.05) and “**” (p < 0.01), *** (p < 0.001), indicated statistically significant between different groups.

## RESULTS

### Bioinformatics analysis of Lnc45

To investigate the potential biological functions of Lnc45, a systematically bioinformatics analysis of Lnc45 was carried out. As a result, using UCSC Genome Browser, we found that Lnc45 was located on chromosome 7qB3 of the mouse genome (Figure 1a). In addition, by PhyloCSF analysis, Lnc45 was proved to be disabled in encoding genes (Figure 1a). By RNAfold webserver, Lnc45 was predicted as a highly folded secondary structure carrying several hairpin loops (Figure 1b) [27]. Moreover, when using TRANSFAC to analyze the associated TFs of Lnc45, we found that a total of 115 TFs were highly related with Lnc45, such as heat shock factor (HSF), macrophage-activating factor (MAF) and interferon regulated factor (IRF) (Figure 2).

### Lnc45 is highly stimulated by different subtypes of IAV strains

To further investigate the expression of Lnc45 induced by CK10, 6TN cells were infected by CK10 virus with a multiplicity of infection (MOI) of 0.5. The qRT-PCR analysis results showed that Lnc45 was up-regulated 3-fold by CK10 virus compared with uninfected cells at 24 h p.i (Figure 3a). In an attempt to define the expression pattern of Lnc45, 6TN cells were infected by CK10 virus and samples were collected at different time points, and the results showed that the expression of Lnc45 reached peak at 28 h p.i (Figure 3b). Moreover, when infected 6TN cells with MOIs of 0.5, 2, 3, 4 or 5, respectively, the expression of Lnc45 gradually increased and showed the highest expression level at an infection dose of 4 MOI (upregulated almost 10-fold) (Figure 3c). We then further investigate whether virus infection-induced highly expression of Lnc45 is specific to CK10 virus or not. Two additional IAV strains were selected, including H1N1 (PR8) and H7N9 (S8). Surprisingly, both S8 and PR8 highly up-regulated the expression of Lnc45; with 10-fold and 3-fold, respectively (Figure 3d). Altogether, these results clearly demonstrated that Lnc45 is highly stimulated by various subtypes of IAV stains.

### Lnc45 is translocated from nuclear to cytoplasm during H5N1 IAV infection

To elucidate the potential role of Lnc45 in influenza virus replication, we then defined the subcellular distributions of Lnc45. Cytoplasmic and nuclear fractions of the 6TN cells were collected, GAPDH and 18S was set as the reference standard for cytoplasm protein, while U6 was used as the reference standard for nuclear protein. The results of RNA FISH revealed that 18S was more abundant in the cytoplasmic fraction of 6TN cells compared with the nuclear fraction (Figure 4a**, upper panel**), while U6 principally localized in nucleus of 6TN cells (Figure 4a**, lower panel**). When determining the localization of Lnc45, we found that Lnc45 distributed mainly in the nucleus of the uninfected cells (Figure 4b and 4c), while some of the Lnc45 transported from nucleus to the cytoplasm after CK10 virus infection (Figure 4b and 4d). Therefore, these results revealed that Lnc45 can translocate from nucleus to cytoplasm during H5N1 IAV infection.

### Forced Lnc45 expression efficiently inhibits viral replication of different subtypes IAV

Since different subtypes of IAV infection can significantly elevate Lnc45 expression, we want to investigate the effect of Lnc45 on viral replication by overexpressing Lnc45. The indirect immunofluorescence assay showed that overexpression of Lnc45 significantly decreased NP expression during CK10 virus infection, suggesting the role of Lnc45 limiting viral replication (Figure 5a and 5c). Moreover, qRT-PCR results confirmed that the expression level of Lnc45 mRNA was increased by 20-fold in 293 T cells (Figure 5b). To confirm these results, we further systematically investigate whether overexpressing Lnc45 affect viral replication in 293 T cells. As a result, the viral growth curves showed that over-expression of Lnc45 significantly inhibited viral replication of the H5N1 (CK10) virus (Figure 5d), H7N9 (S8) virus (Figure 5e) and H1N1 (PR8) virus (Figure 5f) in 293  T cells. Specifically, the viral titer of CK10 was down-regulated 31.63-fold at 48 h p.i, and 37.9-fold at 72 h p.i, respectively (Figure 5d). As for S8 virus, the viral titer was down-regulated 25.12-fold at 60 h p.i, and 39.81-fold at 84 h p.i, respectively (Figure 5e). In addition, the viral titer for PR8 was down-regulated 15.84-fold at 48 h p.i, and 19.95-fold at 60 h p.i, respectively (Figure 5f). Furthermore, qRT-PCR results also showed that overexpression of Lnc45 significantly down-regulated the NP mRNA, cRNA and vRNA level of these viral strains (Figure 5g to Figure 5i). To be noted of, when comparing the expression pattern of NP RNA in different viral stains, we found that the PR8 virus showed the highest NP RNA reduction compared with that of CK10 and S8 virus, with 5-efold for mRNA, 3-fold for cRNA and 2.5-fold for vRNA, respectively (Figure 5i).

In order to verify the antiviral ability of Lnc45, we further determine the effect of over-expression of Lnc45 in viral replication of CK10, S8 and PR8 virus in Hela cells. The results showed that the viral titers of the CK10 virus were significantly down-regulated 19.95-fold at 48 h p.i and 15.84-fold at 72 h p.i (Figure 6a). Moreover, the S8 viral titer was down-regulated 13.8-fold at 36 h p.i (Figure 6b). It’s worth noting that the PR8 viral titer declined significantly at multiple time points compared with empty vector control, including 36 h p.i (3.3-fold), 48 h p.i (6.31-fold) and 60 h p.i (63.1-fold) (Figure 6c). The followed results of qRT-PCR further showed that overexpression of Lnc45 also significantly suppressed the expression of NP mRNA, cRNA and vRNA of the CK10 (Figure 6d), S8 (Figure 6e) and PR8 virus (Figure 6f). More importantly, compared with mRNA and vRNA, the cRNA level showed the highest reduction during different virus infection, with 5-fold for CK10 virus, 6-fold for S8 virus and 2-fold for PR8 virus, respectively (Figure 6d and 6e). Altogether, these results clearly revealed that forced Lnc45 expression efficiently inhibits viral replication of different subtypes of IAV, including H5N1, H7N9 and H1N1 virus strain.

### Abolish of Lnc45 expression highly promotes viral replication of different subtypes IAV

In order to further confirm the antiviral function of Lnc45 during IAV infection, we then analyzed the effect of abolish of Lnc45 expression on viral replication. Lnc45-targeted siRNA (siRNA-700, siRNA-1150, siRNA-700+ siRNA-1150) treatment resulted in a reduction of mRNA level of 90%, 85%, 95% in 6TN cells, respectively (Figure 7a). Therefore, the following results of knockdown of Lnc45 expression were all based on the combination of siRNA-700 and siRNA-1150. The results of qRT-PCR showed that silencing of Lnc45 expression highly upregulated the mRNA, cRNA and vRNA level of NP of different IAV in cellular precipitation, including CK10 (Figure 7b), S8 (Figure 7c) and PR8 virus (Figure 7d). Notably, when comparing the expression pattern of NP RNA in different viral strains, we found that knockdown of Lnc45 has the most obvious effect on the level of CK10 NP mRNA (10-fold), NP cRNA (25-fold) and NP vRNA (20-fold) (Figure 7b). Similar with the result in cellular precipitation, we also observed a significant up-regulation of different NP RNA levels in the culture supernatant of the Lnc45 knockdown cells (Figure 8a, 8b and 8c). Interestingly, knockdown of Lnc45 also has the most obvious effect on the level of CK10 NP RNA in the culture supernatant of the Lnc45 knockdown cells, with NP mRNA increased 6-fold, NP cRNA increased 5-fold and NP vRNA increased 4-fold, respectively (Figure 8a). Moreover, it’s also worth noting that the level of NP mRNA was mostly up-regulated when comparing to cRNA and vRNA in different viral strains (Figure 8a, 8b and 8c). Taken together, these results clearly indicated that knockdown of Lnc45 enhances viral replication of different subtype IAV, further supporting the extensive antivirus activity of Lnc45.

### Forced Lnc45 expression significantly inhibits viral polymerase activity and cell apoptosis

The aforementioned data showed that Lnc45 was involved in extensive antivirus activity. We logically asked whether Lnc45 expression inhibited viral polymerase activity, virus infection-induced cell apoptosis and necrosis. As a result, we found that forced expression of Lnc45 in 293 T cells substantially decreased viral polymerase activity (Figure 9a). Additionally, overexpression of Lnc45 significantly impeded cell apoptosis of the CK10 virus-infected 293 T cells (Figure 9b). However, overexpression of Lnc45 has no effect on cell necrosis during CK10 virus infection (Figure 9c). Altogether, these data suggested that overexpression of Lnc45 significantly inhibits viral polymerase activity and cell apoptosis.

### Forced Lnc45 expression impedes PA and NP nuclear accumulation

To provide mechanistic insight into antiviral action of Lnc45, we further investigate whether Lnc45 affects nuclear aggregation of NP and PA proteins during CK10 virus infection. As shown in Figure 10a and 10b, by IFA assay, we found that over-expression of Lnc45 significantly inhibited PA and NP nuclear accumulation. Moreover, a significant difference in the aggregation of NP and PA proteins in the nucleus was observed at 9 h p.i in overexpressed-Lnc45 cells (Figure 10a, 10b and 10c). Specifically, at 9 h p.i, the PA protein was detected in the nucleus of 80% of the mock control cells, whereas only 25% of cells in the over-expression group showed PA nuclear localization (Figure 10d). Similarly, NP protein was detected in the nucleus of 95% of the mock control cells, whereas only 25% of cells in the over-expression group showed PA nuclear localization (Figure 10d). Therefore, these results directly indicated that over-expression of Lnc45 impedes nuclear aggregation of viral NP and PA proteins in 293 T cells.

### The stem ring arms mainly determine the antiviral effect of Lnc45

It is well known that the diverse functions of lncRNA are highly associated with their ability of folding into thermodynamically stable secondary and higher-order structures [28]. We then further dissected the functional structures associated with the antiviral ability of Lnc45. Twelve truncation mutants were successfully constructed according to the predicted secondary structure of Lnc45 through RNAfold analysis (Figure 11a and 11b). More specifically, according to the location of stem-loop arm, Lnc45 was divided into Mutant A to Mutant L. Additionally, there are several stem-loop arms at each end of Lnc45’s secondary structure. By RT-PCR, the ectopic expression of these mutants was successfully confirmed (Figure 11c). Further functional experiments concerning the effect of these mutants on viral replication revealed that the stem ring arms mainly determine the antiviral effect of Lnc45 (Figure 11d). Specifically, the RNA sequences of stem loops in Lnc45, including 1–190 nt, 330–570 nt, 780–830 nt, 1310–1430 nt, 1515–1668 nt and 1714–1890 nt may form a spatial structure that is essential for its antiviral function.

## DISCUSSION

Recently, accumulated studies have shown that IAV infection can induce a huge number of differentially expressed lncRNA in host cells [11,17,19,20,29]. LncRNA represents as a class of host factors, which is important for antiviral strategies [30]. As a large number of lncRNA have been discovered, many of the associated biofunctions have been successfully determined. Revealing the function of lncRNA in the process of influenza virus infection and replication is of great significance for elucidating the pathogenesis of influenza virus [31]. In this study, we demonstrated that a functional lncRNA, Lnc45, which was correlated with multiple transcription factors (TFs), including IRF, MAF and HSF (Figure 2), had a profound influence on viral replication of various subtype of IAV (Figure 5 **to** Figure 8). Furthermore, Lnc45 also acts as an important regulatory molecule, including inhibits viral polymerase activity and cell death (Figure 9), restrains nuclear aggregation of viral NP and PA proteins during influenza virus infection (Figure 10). Notably, multiple functional domains of Lnc45 which may form a stable spatial structure are required for its antiviral activity, mainly the stem ring arms of Lnc45 (Figure 11).

TFs are a type of proteins that combine with the promoter region of genes to turn on the transcription process, and plays an essential role in regulating signaling cascades during pathogen infection [32,33]. Interestingly, lncRNA emerged as crucial regulator in gene expression and is highly associated with TFs [4]. In this study, we found that Lnc45 associated with 115 TFs, including IRF, MAF, and HSF (Figure 2). Importantly, several TFs are associated with the innate immune response such as MAF, IRF, and AIRE (autoimmune regulator). MAF family genes are a class of TFs that play an important role in tumorigenesis, immune system, insulin production, cell differentiation, and apoptosis [34]. In addition, MAF indirectly regulates viral replication during influenza virus infection via interaction with MAPK (mitogen-activated protein kinase) and AKT/mTOR signaling pathways [35]. IRF, is a kind of transcriptional factors plays a critical and diverse role, mainly include establish a close link between microbial signatures and the IFNs representative innate immune responses [36,37]. Although the mechanism underlying the highly activation of Lnc45 by various subtype influenza virus remains elusive, it is highly possible that these important TFs are likely contributed to particular pathways that are involved in activation of Lnc45 after sensing influenza virus infection. Future studies are still needed to verify whether the correlation of the TFs and Lnc45 is linked to the Lnc45-mdieated antiviral ability as well as elucidate the potential associated molecular mechanism.

Currently, the roles of lncRNA during virus infection have been progressively unveiled. Multifarious of viruses, including coronavirus, IAV, enterovirus, HCV, rabies virus, HIV, hepatitis B virus (HBV), Japanese encephalitis virus (JEV), encephalomyocarditis virus (EMCV), have been demonstrated to stimulate various of lncRNA transcription in different host cells [8,21,38–40]. For example, Ma *et al.* found that lncRNA NEAT1 that was stimulated by Hantan virus (HTNV) via RIG-I-IRF7 pathway highly promotes IFN expression through facilitating RIG-I and DEXD/H box helicase 60 (DDX60) expression [38]. Lnc45 is also highly up-regulated by different subtypes IAVs (Figure 3d). It is worth noting that forced expression or silencing of Lnc45 resulted in a significantly decreased or enhanced viral replication of different subtype virus, suggesting the broad-spectrum antiviral ability of Lnc45. Currently, other functional lncRNA such as Lnc-ISG20, LncRNA-PAAN (PA-associated noncoding RNA), LncRNA-155 and VIN also have been identified as important regulators during IAV infection [11,15,16,20]. For example, Lnc-ISG20 suppresses IAV replication via up-regulating ISG20 production, while LncRNA-PAAN promotes IAV replication by enhancing influenza virus RNA polymerase activity [11,20]. LncRNA-155 enhances IAV-mediated innate immune response through elevating IFN-β expression and reinforcing the associated type I IFN signaling pathway, including augmenting the production of several critical ISGs. In addition, the large intergenic ncRNAs, VIN, promotes replication of influenza viruses without interference from interferon or interferon stimulants, and can be activated by a variety of influenza viruses (including H1N1, H3N2, and H7N7 influenza viruses) and vesicular stomatitis viruses (VSV) [15,16]. We here demonstrated that Lnc45 is not only induced by H5N1 IAV, but also other subtype IAVs, including H7N9 and H1N1 influenza virus (Figure 3d). More importantly, we also showed that Lnc45 has a broad inhibitory effect on viral replication of H5N1, H7N9 and H1N1 subtype virus. Currently, we are exploring the association of Lnc45 with host innate immunity and elucidating the potential role of the close interaction between viral proteins and Lnc45 in regulating IAV infection.

In this study, we found that the antiviral activity of Lnc45 was strain-dependent characterized by stronger suppressive effect observed in some IAV strains than others. For example, Lnc45 exerts a more potent inhibitory effect in CK10-infected 293 T cells. Specifically, the viral titer of CK10 virus decreased by 31.63-fold at 48 h p.i, and 37.9-fold at 72 h p.i, respectively (Figure 5d), while lower reduction in virus titers were detected for S8 and PR8 viruses (Figure 5e and 5f). As for the viral titers in Hela cells, it seems likely that Lnc45 exerts more prominent antiviral effects against PR8 virus. For example, Lnc45 inhibits PR8 virus replication at multiple time points, and the viral titer of PR8 dropped by 3.3-fold at 36 h p.i, 6.31-fold at 48 h p.i and 63.1-fold at 60 h p.i, respectively (Figure 6c). By contrast, the viral titers of CK10 reduced 19.95-fold at 48 h p.i and 15.84-fold at 72 h p.i (Figure 6a), while S8 viral titer only declined 13.8-fold at 36 h p.i (Figure 6b). Interestingly, our results also showed that Lnc45 inhibits viral replication probably by impeding polymerase activity (Figure 9) and nuclear accumulation efficiency of NP and PA (Figure 10). Therefore, we hypothesized that the strain-specific antiviral effect seen for Lnc45 may be related to the discrepancy of Lnc45 in inhibiting polymerase activity and nuclear accumulation efficiency of NP and PA among different virus strains. Furthermore, Lnc45 may inhibit viral replication by interacting with viral NP and PA to further regulate polymerase activity and nuclear accumulation efficiency. Future studies are required to verify these assumptions.

At present, there are mainly two reported functional patterns of lncRNA, one is modulating gene expression in the nucleus [41], another is affecting signal transduction or assimilating microRNAs in the cytoplasm [42–45]. For instance, LncRNA Morrbid (myeloid RNA regulator of Bim-induced death), which predominately localizes in the nucleus, modulates Bim gene transcription to control the lifespan of the short-lived myeloid cell, such as eosinophils, neutrophils and monocytes [41]. However, lncRNA also regulates cellular differentiation and function through close interconnection with signaling transduction molecules in the cytoplasm [42,46]. Taking virus-induced lncRNA-ACOD1 (aconitate decarboxylase 1) as an example, lncRNA-ACOD1 that is independent of type I interferon mainly located in cytoplasm can facilitate viral replication through associating tightly with metabolic enzyme glutamic-oxaloacetic transaminase (GOT2) [42]. Moreover, Carrieri *et al*. found that the localization and function of antisense Ubiquitin carboxy-terminal hydrolase L1 (Uchl1) is regulated by mTOR pathway. Inhibition of mTORC1 results in up-regulation of Uchl1 and is associated with the shuttling of Uchl1 from the nucleus to the cytoplasm [46]. In this study, we identified that Lnc45 was mainly located in nuclear before virus infection, while it translocated to cytoplasm after H5N1 influenza virus infection (Figure 4a and 4b). We supposed that the transportation of Lnc45 from nuclear to cytoplasm may be associated with its antiviral function, which is fundamental for its role in regulating the translation of some important functional genes or the expression of some crucial proteins related with influenza viral replication.

Previous studies have demonstrated that the ability of polymerase protein accumulation in nuclear highly correlates with the virulence of influenza virus [47–51]. For example, Chen *et al*. found that K91R and K198R disrupts cellular location of NP and decreases viral polymerase activity and subsequently contribute to H5N1 viral virulence in mammals [47]. PA K237E mutation increased PA nuclear accumulation in DEFs and contributed to the enhanced virulence of the highly pathogenic H5N1 virus in ducks [50]. In addition, Giese *et al*. also found that the enhanced transportation of PB2 from cytoplasm to the nucleus is highly associated with the enhanced virulence of the H7N7 virus in a mouse model [51]. In this study, we found that overexpression of Lnc45 suppressed the nuclear accumulation of PA and NP (Figure 10), and we hypothesized that this dynamic distribution character of Lnc45 may also relate to its antiviral effect (Figure 5 to Figure 8). The influenza viral RNP complex is composed of PB2, PB1, NP, and PA. Some previous studies have demonstrated that specific mutations in the RNP complex associated with viral replication also affect viral polymerase activity and the nuclear accumulation ability of the RNP component [50,52–54]. In this study, we found that the antiviral capability of Lnc45 is also associated with its ability to inhibit polymerase activity and nuclear accumulation of NP and PA. However, currently, we cannot exclude the probability that the lower levels of NP and PA nuclear accumulation may also relate to the reduced viral polymerase activity caused by Lnc45 expression. Therefore, further studies are needed to verify this possibility in our future study.

In conclusion, this is the first study to describe the broad antiviral role of Lnc45 during IAV infection. We have provided direct evidence that Lnc45 is highly stimulated by various subtypes of IAV and acts as a broad-spectrum antiviral factor. More importantly, we also demonstrated that the antiviral capability attributed to Lnc45 may be associated with its ability to inhibit polymerase activity and NP and PA nuclear accumulation mainly via its stem ring arms. Further study aimed at elucidating the potential mechanism of the antiviral effect of Lnc45 is highly needed, which may include investigating the potential contribution of the Lnc45-mediated-cellular response as to the universal defense against influenza virus infection and determining the exact relationship between Lnc45 distribution and its antiviral activities.

## Supplementary Material

Supplemental Material

## Data Availability

Data sharing is not applicable to this article as no new data were created or analyzed in this study

## References

[1] Long JS, Mistry B, Haslam SM, et al. Host and viral determinants of influenza A virus species specificity [J]. Nat Rev Microbiol. 2019;17(2):67–81. 30487536 10.1038/s41579-018-0115-z

[2] Salomon R, Webster RG. The influenza virus enigma [J]. Cell. 2009;136(3):402–410. 19203576 10.1016/j.cell.2009.01.029 PMC2971533

[3] Kotzin JJ, MowelL WK, Henao-mejia J. Viruses hijack a host lncRNA to replicate [J]. Science. 2017;358(6366):993–994. 29170219 10.1126/science.aar2300

[4] Long Y, Wang X, Youmans DT, et al. How do lncRNAs regulate transcription? [J]. Sci Adv. 2017;3(9):eaao2110. 28959731 10.1126/sciadv.aao2110 PMC5617379

[5] Carpenter S, Fitzgerald KA. Transcription of inflammatory genes: long noncoding RNA and beyond [J]. J Interferon Cytokine Res. 2015;35(2):79–88. 25250698 10.1089/jir.2014.0120 PMC4312878

[6] Ding Y.Z, Zhang Z.W, Liu Y.L, Ding Y Z, Zhang Z W, Liu Y L, et al. Relationship of long noncoding RNA and viruses [J]. Genomics. 2016;107(4):150–154. 26826341 10.1016/j.ygeno.2016.01.007

[7] Zhang Q, Jeang KT. Long non-coding RNAs (lncRNAs) and viral infections [J]. Biomed Pharmacother. 2013;3(1):34–42. 23645970 10.1016/j.biomed.2013.01.001 PMC3641704

[8] Carnero E, Barriocanal M, Prior C, et al. Long noncoding RNA EGOT negatively affects the antiviral response and favors HCV replication [J]. EMBO Rep. 2016;17(7):1013–1028. 27283940 10.15252/embr.201541763 PMC4931568

[9] Imam H, Bano AS, Patel P, et al. The lncRNA NRON modulates HIV-1 replication in a NFAT-dependent manner and is differentially regulated by early and late viral proteins [J]. Sci Rep. 2015;5:8639. doi: 10.1038/srep08639. PMC4345339 25728138

[10] Qian X, Xu C, Zhao P, et al. Long non-coding RNA GAS5 inhibited hepatitis C virus replication by binding viral NS3 protein [J]. Virology. 2016;492:155-65. 10.1016/j.virol.2016.02.020 26945984

[11] Wang J, Wang Y, Zhou R, et al. Host Long Noncoding RNA lncRNA-PAAN Regulates the Replication of Influenza A Virus [J]. Viruses. 2018;10(6):330. 10.3390/v10060330 PMC6024364 29914164

[12] Ouyang J, Hu J, Chen JL. lncRNAs regulate the innate immune response to viral infection [J]. Wiley Interdiscip Rev RNA. 2016;7(1):129–143. 26667656 10.1002/wrna.1321 PMC7169827

[13] Fukushima K, Kida H. A host lncRNA regulates the innate immune response to an RNA virus [J]. Cell Mol Immunol. 2019;16(10):841–842. 31467412 10.1038/s41423-019-0280-7 PMC6804522

[14] Lin H, Jiang M, Liu L, et al. The long noncoding RNA Lnczc3h7a promotes a TRIM25-mediated RIG-I antiviral innate immune response [J]. Nat Immunol. 2019;20(7):812–823. 31036902 10.1038/s41590-019-0379-0

[15] Maarouf M, Chen B, Chen Y, et al. Identification of lncRNA-155 encoded by MIR155HG as a novel regulator of innate immunity against influenza A virus infection [J]. Cell Microbiol. 2019;21(8):e13036. 31045320 10.1111/cmi.13036

[16] Winterling C, Koch M, Koeppel M, et al. Evidence for a crucial role of a host non-coding RNA in influenza A virus replication [J]. RNA Biol. 2014;11(1):66–75. 24440876 10.4161/rna.27504 PMC3929426

[17] Wang Q, Zhang D, Feng W, et al. Long noncoding RNA TSPOAP1 antisense RNA 1 negatively modulates type I IFN signaling to facilitate influenza A virus replication [J]. J Med Virol. 2019. doi: 10.1002/jmv.25483. 30968963

[18] Hadjicharalambous M, Lindsay M. Long Non-Coding RNAs and the Innate Immune Response [J]. Noncoding RNA. 2019;5(2):34. 10.3390/ncrna5020034 PMC6630897 31010202

[19] Pan Q, Zhao Z, Liao Y, et al. Identification of an Interferon-Stimulated Long Noncoding RNA (LncRNA ISR) Involved in Regulation of Influenza A Virus Replication [J]. Int J Mol Sci. 2019;20(20):5118. 10.3390/ijms20205118 PMC6829313 31623059

[20] ChaiI W, Li J, Shangguan Q, et al. Lnc-ISG20 Inhibits Influenza A Virus Replication by Enhancing ISG20 Expression [J]. J Virol. 2018;92(16). DOI:10.1128/JVI.00539-18. PMC6069214 29899085

[21] Ouyang J, Zhu X, Chen Y, et al. NRAV, a long noncoding RNA, modulates antiviral responses through suppression of interferon-stimulated gene transcription [J]. Cell Host Microbe. 2014;16(5):616–626. 25525793 10.1016/j.chom.2014.10.001 PMC7104942

[22] Zhang Y, Cao X. Long noncoding RNAs in innate immunity [J]. Cell Mol Immunol. 2016;13(2):138–147. 26277893 10.1038/cmi.2015.68 PMC4786632

[23] Hu J, Hu Z, Wang X, et al. Deep sequencing of the mouse lung transcriptome reveals distinct long non-coding RNAs expression associated with the high virulence of H5N1 avian influenza virus in mice [J]. Virulence. 2018;9(1):1092–1111. 30052469 10.1080/21505594.2018.1475795 PMC6086314

[24] Hu J, Hu Z, Song Q, et al. The PA-Gene-Mediated Lethal Dissemination and Excessive Innate Immune Response Contribute to the High Virulence of H5N1 Avian Influenza Virus in Mice [J]. J Virol. 2012;87(5):2660–2672. 23255810 10.1128/JVI.02891-12 PMC3571398

[25] Hu J, Mo Y, Wang X, et al. PA-X decreases the pathogenicity of highly pathogenic H5N1 influenza A virus in avian species by inhibiting virus replication and host response [J]. J Virol. 2015;89(8):4126–4142. 25631083 10.1128/JVI.02132-14 PMC4442343

[26] Labarre DD, Lowy RJ. Improvements in methods for calculating virus titer estimates from TCID50 and plaque assays [J]. J Virol Methods. 2001;96(2):107–126. 11445142 10.1016/s0166-0934(01)00316-0

[27] Washietl S, Hofacker IL, Lukasser M, et al. Mapping of conserved RNA secondary structures predicts thousands of functional noncoding RNAs in the human genome [J]. Nat Biotechnol. 2005;23(11):1383–1390. 16273071 10.1038/nbt1144

[28] Rivas E, Clements J, Eddy SR. A statistical test for conserved RNA structure shows lack of evidence for structure in lncRNAs [J]. Nat Methods. 2017;14(1):45–48. 27819659 10.1038/nmeth.4066 PMC5554622

[29] Wang J, Zhang Y, Li Q. Influenza Virus Exploits an Interferon-Independent lncRNA to Preserve Viral RNA Synthesis through Stabilizing Viral RNA Polymerase PB1 [J]. Cell Rep. 2019;27(11):3295–304.e4. 31189112 10.1016/j.celrep.2019.05.036

[30] Carpenter S, Fitzgerald KA. Cytokines and Long Noncoding RNAs [J]. Cold Spring Harb Perspect Biol. 2018;10(6):a028589. 10.1101/cshperspect.a028589 PMC5983188 28716885

[31] Ma Y, Ouyang J, Wei J, et al. Involvement of Host Non-Coding RNAs in the Pathogenesis of the Influenza Virus [J]. Int J Mol Sci. 2016;18(1):39. 10.3390/ijms18010039 PMC5297674 28035991

[32] WingenderR E, Chen X, Hehl R, et al. TRANSFAC: an integrated system for gene expression regulation [J]. Nucleic Acids Res. 2000;28(1):316–319. 10592259 10.1093/nar/28.1.316 PMC102445

[33] Dykes IM, Transcriptional EMANUELIC. Post-transcriptional Gene Regulation by Long Non-coding RNA [J]. Genomics Proteomics Bioinformatics. 2017;15(3):177–186. 28529100 10.1016/j.gpb.2016.12.005 PMC5487525

[34] Zhang C, Guo ZM. Multiple functions of Maf in the regulation of cellular development and differentiation [J]. Diabetes Metab Res Rev. 2015;31(8):773–778. 26122665 10.1002/dmrr.2676 PMC5042042

[35] Brundage ME, Tandon P, Eaves DW, et al. MAF mediates crosstalk between Ras-MAPK and mTOR signaling in NF1 [J]. Oncogene. 2014;33(49):5626–5636. 24509877 10.1038/onc.2013.506 PMC4127377

[36] Chiang HS, Liy HM. The Molecular Basis of Viral Inhibition of IRF- and STAT-Dependent Immune Responses [J]. Front Immunol. 2018;9:3086. 10.3389/fimmu.2018.03086 PMC6332930 30671058

[37] Battistini A. Interferon regulatory factors in hematopoietic cell differentiation and immune regulation [J]. J Interferon Cytokine Res. 2009;29(12):765–780. 19929577 10.1089/jir.2009.0030

[38] Ma H, Han P, Ye W, et al. The Long Noncoding RNA NEAT1 Exerts Antihantaviral Effects by Acting as Positive Feedback for RIG-I Signaling [J]. J Virol. 2017; 91(9):e02250-16 . 10.1128/JVI.02250-16 PMC5391460 28202761

[39] Kambara H, Niazi F, Kostadinova L, et al. Negative regulation of the interferon response by an interferon-induced long non-coding RNA [J]. Nucleic Acids Res. 2014;42(16):10668–10680. 25122750 10.1093/nar/gku713 PMC4176326

[40] Nishitsuji H, Ujino S, Yoshio S, et al. Long noncoding RNA #32 contributes to antiviral responses by controlling interferon-stimulated gene expression [J]. Proc Natl Acad Sci U S A. 2016;113(37):10388–10393. 27582466 10.1073/pnas.1525022113 PMC5027408

[41] Kotzin JJ, Spencer SP, Mccright SJ, et al. The long non-coding RNA Morrbid regulates Bim and short-lived myeloid cell lifespan [J]. Nature. 2016;537(7619):239–243. 27525555 10.1038/nature19346 PMC5161578

[42] Wang P, Xu J, Wang Y, et al. An interferon-independent lncRNA promotes viral replication by modulating cellular metabolism [J]. Science (New York, NY). 2017; 358(6366): 1051–1055. 10.1126/science.aao0409 29074580

[43] Lin A, Li C, Xing Z, et al. The LINK-A lncRNA activates normoxic HIF1α signalling in triple-negative breast cancer [J]. Nat Cell Biol. 2016;18(2):213–224. 26751287 10.1038/ncb3295 PMC4791069

[44] Tay Y, Kats L, Salmena L, et al. Coding-Independent Regulation of the Tumor Suppressor PTEN by Competing Endogenous mRNAs [J]. Cell. 2011;147(2):344–357. 22000013 10.1016/j.cell.2011.09.029 PMC3235920

[45] Wang P, Xue Y, Han Y, et al. The STAT3-Binding Long Noncoding RNA lnc-DC Controls Human Dendritic Cell Differentiation [J]. Science. 2014;344(6181):310–313. 24744378 10.1126/science.1251456

[46] Carrieri C, Cimatti L, Biagioli M, et al. Long non-coding antisense RNA controls Uchl1 translation through an embedded SINEB2 repeat [J]. Nature. 2012;491(7424):454–457. 23064229 10.1038/nature11508

[47] Chen L, Wang C, Luo J, et al. Amino Acid Substitution K470R in the Nucleoprotein Increases the Virulence of H5N1 Influenza A Virus in Mammals [J]. Front Microbiol. 2017;8:1308. doi: 10.3389/fmicb.2017.01308 PMC5504190 28744280

[48] Kashwagi T, Leung BW, Deng T, et al. The N-terminal region of the PA subunit of the RNA polymerase of influenza A/HongKong/156/97 (H5N1) influences promoter binding [J]. PLoS One. 2009;4(5):e5473. 19421324 10.1371/journal.pone.0005473 PMC2674210

[49] The FODORE. RNA polymerase of influenza a virus: mechanisms of viral transcription and replication [J]. Acta Virol. 2013;57(2):113–122. 23600869 10.4149/av_2013_02_113

[50] Hu J, Hu Z, Mo Y, et al. The PA and HA gene-mediated high viral load and intense innate immune response in the brain contribute to the high pathogenicity of H5N1 avian influenza virus in mallard ducks [J]. J Virol. 2013;87(20):11063–11075. 23926340 10.1128/JVI.00760-13 PMC3807287

[51] Giese S, Ciminski K, Bolte H, et al. Role of influenza A virus NP acetylation on viral growth and replication [J]. Nat Commun. 2017;8(1):1259. 29097654 10.1038/s41467-017-01112-3 PMC5668263

[52] Gabriel G, Dauber B, Wolff T, et al. The viral polymerase mediates adaptation of an avian influenza virus to a mammalian host [J]. Proc Natl Acad Sci U S A. 2005;102(51):18590–18595. 16339318 10.1073/pnas.0507415102 PMC1317936

[53] Song J, Feng H, Xu J, et al. The PA Protein Directly Contributes to the Virulence of H5N1 Avian Influenza Viruses in Domestic Ducks [J]. J Virol. 2010;85(5):2180–2188. 21177821 10.1128/JVI.01975-10 PMC3067757

[54] Arai Y, Kawashita N, Ibrahim MS, et al. PB2 mutations arising during H9N2 influenza evolution in the Middle East confer enhanced replication and growth in mammals [J]. PLoS Pathog. 2019;15(7):e1007919. 31265471 10.1371/journal.ppat.1007919 PMC6629154

