# Supplementary material for: Long noncoding RNA#45 exerts broad inhibitory effect on influenza a virus replication via its stem ring arms
Source: Virulence. 2021 Sep 14;12(1):2443–60. doi: 10.1080/21505594.2021.1975494 (PMC8451462; doi:10.1080/21505594.2021.1975494)
Supplement: Supplemental Material [file KVIR_A_1975494_SM6769.docx]

**Table S1** Primers used for truncation mutant plasmids construction

| Primers | Primer sequences (5′–3′) |
| --- | --- |
| Mutant-A-F | CCGCTCGAGTCTCTCTCTCTCTCTCTCTCTCTCTCT |
| Mutant-A-R | GCTCTAGAGGTTTTTTCAAGACAGAGTTTCTCT |
| Mutant-B-F | CCGCTCGAG GATGAGCAGTTCACGTCTATATCTG |
| Mutant-B-R | GCTCTAGAGGTTTTTTCAAGACAGAGTTTCTCT |
| Mutant-C-F | CCGCTCGAG CCTGTAATGTTTGTACAGCAGTCTT |
| Mutant-C-R | GCTCTAGAGGTTTTTTCAAGACAGAGTTTCTCT |
| Mutant-D-F1 | CCGCTCGAG GTAGGCTTCAAGGATGGGGG |
| Mutant-D-R1 | CTATATTTCCCTTGA-ATCACTCTCCACAAGGGGGC |
| Mutant-D-F2 | CTTGTGGAGAGTGAT-TCAAGGGAAATATAGCCCAC |
| Mutant-D-R2 | GCTCTAGAGGTTTTTTCAAGACAGAGTTTCTCT |
| Mutant-E-F1 | CCGCTCGAGGTAGGCTTCAAGGATGGGG |
| Mutant-E-R1 | GGGCTAAATTCTGCT-GATATAGACGTGAACTGCTCATCAC |
| Mutant-E-F2 | GTTCACGTCTATATC-AGCAGAATTTAGCCCAGGTT |
| Mutant-E-R2 | GCTCTAGAGGTTTTTTCAAGACAGAGTTTCTCT |
| Mutant-F-F1 | CCGCTCGAGGTAGGCTTCAAGGATGGGGG |
| Mutant-F-R1 | GTTTCTTGTCTATGT-AAATTCTGCTCTTCCAGGGG |
| Mutant-F-F2 | GGAAGAGCAGAATTT-ACATAGACAAGAAACTGTCATCAAG |
| Mutant-F-R2 | GCTCTAGAGGTTTTTTCAAGACAGAGTTTCTCT |
| Mutant-G-F1 | CCGCTCGAGGTAGGCTTCAAGGATGGGG |
| Mutant-G-R1 | AGAGAGAGAGAGAGA-CTTATGGCTGGTGGGCTATA |
| Mutant-G-F2 | CCCACCAGCCATAAG-TCTCTCTCTCTCTCTCTCTCTCTCT |
| Mutant-G-R2 | GCTCTAGAGGTTTTTTCAAGACAGAGTTTCT |
| Mutant-H-F | CCGCTCGAGGTAGGCTTCAAGGATGGGGGC |
| Mutant-H-R | GCTCTAGATGGCACACACCTTTAATCCCAG |
| Mutant-I-F1 | CCGCTCGAGGTAGGCTTCAAGGATGGGGG |
| Mutant-I-R1 | TGCTATCCACCATGG-CTGCTCCTCAGGACGGTG |
| Mutant-I-F2 | CGTCCTGAGGAGCAG-CCATGGTGGATAGCACTCTTC |
| Mutant-I-R2 | GCTCTAGAGGTTTTTTCAAGACAGAGTTTCTCT |
| Mutant-J-F1 | CCGCTCGAGGTAGGCTTCAAGGATGGGGG |
| Mutant-J-R1 | CAGAGGTAACAAGGG-CTGCTCCTCAGGACGGTG |
| Mutant-J-F2 | CGTCCTGAGGAGCAG-CCCTTGTTACCTCTGAAGGAG |
| Mutant-J-R2 | GCTCTAGAGGTTTTTTCAAGACAGAGTTTCTCT |
| Mutant-K-F1 | CCGCTCGAGGTAGGCTTCAAGGATGGGGG |
| Mutant-K-R1 | TGTTCTCTTTTCCTT-GCGCAGCTCTGTTCTGAGTC |
| Mutant-K-F2 | AGAACAGAGCTGCGC-AAGGAAAAGAGAACACAAGAGAAAG |
| Mutant-K-R2 | GCTCTAGAGGTTTTTTCAAGACAGAGTTTCTCT |
| Mutant-L-F1 | CCGCTCGAGGTAGGCTTCAAGGATGGGG |
| Mutant-L-R1 | TTTTGTTTTTTGTTT-CCCTCTGCTTCCGATCCTT |
| Mutant-L-F2 | ATCGGAAGCAGAGGG-AAACAAAAAACAAAAACAAACCA |
| Mutant-L-R2 | GCTCTAGAGGTTTTTTCAAGACAGAGTTTCT |
